# Validation of 4 Estimating Methods to Evaluate 24-h Urinary Sodium Excretion: Summer and Winter Seasons for College Students in China

**DOI:** 10.3390/nu14132736

**Published:** 2022-06-30

**Authors:** Weiyi Gong, Yuxia Ma, Zechen Zhang, Jufeng Liang, Jiguo Zhang, Gangqiang Ding

**Affiliations:** 1 Key Laboratory of Trace Elements Nutrition of National Health Commission, National Institute for Nutrition and Health, Chinese Center for Disease Control and Prevention, Beijing 100050, China; gongweiyi@ninh.chinacdc.cn; 2 Hebei Key Laboratory of Environment and Human Health, Department of Nutrition and Food Hygiene, School of Public Health, Hebei Medical University, Shijiazhuang 050017, China; mayuxia@hebmu.edu.cn (Y.M.); 13613319942@163.com (Z.Z.); liangjufeng@hebmu.edu.cn (J.L.)

**Keywords:** sodium, salt, urine, methods, assessment

## Abstract

Twenty-four-hour urine sample collection is regarded as the gold standard for sodium intake evaluation, but the implementation can be difficult. The objective was to validate and evaluate the accuracy and feasibility of estimating sodium intake by four methods. A group of 268 healthy volunteers aged 18–25 years was enrolled in this study. Twenty-four-hour urine samples as well as timed (morning, afternoon, evening, and overnight) urine samples were randomly collected in summer and winter. The sodium intake was estimated by four published methods—Kawasaki, INTERSALT, Tanaka, and Sun’s. The consistencies between estimated sodium intake and real measured values of 24-h urinary sodium excretion were compared by Bland–Altman plots in each of the methods. The 24-h urinary sodium analysis result indicated that average daily sodium intake was 3048.4 ± 1225.9 mg in summer and 3564.7 ± 1369.9 mg in winter. At the population level, the bias (estimated value-measured value) was the least with the INTERSALT method with afternoon (−39.7 mg; 95%CI: −164.7, 85.3 mg) and evening (−43.5 mg; 95%CI: −166.4, 79.5 mg) samples in summer. In winter, the Kawasaki method (162.1 mg; 95%CI: 13.5, 310.7 mg) was superior to others. Estimation of sodium intake using the four methods is affected by the time and temperature. In summer, the INTERSALT method provides the best estimation of the population’s mean sodium intake. The Kawasaki method is superior to other methods in winter.

## 1. Introduction

With the development of the economy, the number of patients with cardiovascular disease (CVD) is growing rapidly. Hypertension is the major cause of cardiovascular disease, and high sodium intake is closely related to hypertension [1,2]. Evidence shows that a moderate reduction in sodium consumption can reduce the incidence of cardiovascular disease and medical costs [3]. Besides, studies have also shown that a high sodium diet is one of the risk factors for kidney disease, osteoporosis, and even gastric cancer [4,5]. Therefore, reducing sodium intake has become an important measure to prevent and control non-communicable diseases worldwide [6]. The WHO recommends that adults consume less than 5 g of salt (or 2 g of sodium) per day [7,8]. However, the average daily intake of salt in China is 9.3 g/day/person in 2015 [9], which is far more than the recommended value. The GBDs and CNNSs indicate that a high intake of sodium was the leading dietary risk for deaths, DALYs, and cardiometabolic mortality in China [10,11]. It is vital to accurately evaluate the sodium intake to support efforts to reduce sodium intake.

There are several methods for estimating sodium intake, including dietary survey, 24-h urine collection, and casual (spot) urine sodium measurements. The sodium intake is mainly excreted by the kidney, so 24-h urine collection is considered the gold standard for estimating sodium intake [12,13,14,15,16]. This method is relatively accurate and reliable. However, there are certain limitations in practical applications, which bring psychological burden to the participants as well as difficulties in ensuring the sample integrity. Dietary survey tends to underestimate sodium intake because of the emergence of much more processed food and eating out of home. Recently, some scholars have created the “One-Week Salt Estimation Method”, which is easy to operate and useful in identifying the sources of salt in the diet [17]. However, the method lasts for a whole week and is poorly compliant for most families, making it difficult to be promoted to the whole population as a method to estimate salt intake. Relatively, collecting a spot or casual urine specimen is much more convenient than others. In previous studies, some predictive methods were developed to estimate population’s mean and individual 24-h urinary sodium excretion, of which the most common methods were Kawasaki, INTERSALT, and Tanaka [18,19,20]. There is also one estimated method created by Ningling Sun et al. based on the Chinese sample [21], we call it the Sun’s method here. The validation of these four methods in the representative Chinese young population is limited.

The purpose of our study is to validate and evaluate the accuracy and feasibility of estimating sodium intake by these four approaches in college students. In addition, considering the effect of air temperature on perspiration, this study conducted a comparative study in summer and winter to analyze the difference.

## 2. Materials and Methods

### 2.1. Participants

A cross-sectional survey was conducted in June and November, respectively. A group of 274 healthy volunteers aged from 18 to 25 years old from Hebei Medical University was enrolled in this study. Finally, a total of 268 (138 male and 130 female) participants completed 24-h urine collection in both summer and winter. The study has been approved by Human Trial Ethic Inspection Committee, National Institute for Nutrition and Health, Chinese Center for Disease and Prevention (No: 2017-004). All participants provided written informed consent in this study. The inclusion criteria were: (1) Willing and insisting on completing the survey in two seasons, summer and winter, (2) willing and able to collect 24-h urine, (3) for women, not be in menstruation (if in the menstrual period, postpone 24-h urine collection until the end of the menstrual period), (4) avoiding strenuous exercise during the urine collection period. The exclusion criteria were: (1) The 24-h urine volume < 500 mL, (2) the urine collection time < 20 h, (3) more than one time the urine collection missing or large overflow [22].

### 2.2. Data Collection

The common information was collected through a questionnaire, including name, student ID, age, and gender. Height, weight, waist circumference (WC), and blood pressure (BP) were measured in all participants. Then, the spot time and 24-h urine samples were collected.

### 2.3. 24-h Urine Collection and Measurements

All participants were trained to collect 24-h urine samples themselves. Each time the urine was collected separately in standard containers that were provided, and the participant recorded the name, student number, and the time of the collection, then put it in a special location. The first urine of the day was discarded, and all urine over the following 24 h was collected. The spot urine samples were as follows: Morning (8–12 o’clock), afternoon (12–18 o’clock), evening (18–24 o’clock), overnight (the first urine sample in the next morning). Four spot time urine samples were randomly selected for each participant. Then, a 2 mL aliquot was taken from each of them. Finally, all urine from the same participant was carefully mixed. The total volume of the collection was measured (including the 8 mL we took before), and a 2 mL aliquot was taken. All of the urine samples were stored at −80 °C refrigerator for testing. Urine sodium (Na), potassium (K), and creatinine (Cr) were tested. 

### 2.4. Estimation of 24-h Sodium Excretion from Spot Urine Samples

Measured 24-h urinary sodium excretion (using 24-h urine sample) was calculated as follows: The sodium concentration (mmol/L) × total volume (L/day) × molecular weight of Na^+^ (23 Na^+^). Predictive 24-h urinary sodium excretion from spot urinary sodium concentration used four previously published methods—Kawasaki, INTERSALT, Tanaka, and Sun’s method [18,19,20,21]. These predictive methods are listed in Supplementary Materials.

### 2.5. Statistical Analysis

Continuous variables were presented as mean ± standard deviation. Paired *t*-test was used for comparison between measured values by 24-h urine collection in summer and winter. The group bias in predicted 24-h urine excretion was calculated as the difference between estimated values and measured values for each participant. Then, mean of these differences was calculated. We used Bland–Altman plot to illustrate the relative individual differences between estimated values and measured values by 24-h urine collection. Pearson correlation was used to describe correlation. All analyses were conducted using SAS version 9.4 (SAS Institute Inc., Cary, NC, USA), with the significance level set at a 2-sided *p* < 0.05.

## 3. Results

### 3.1. Basic Characteristics

A total of 268 participants (138 men and 130 women) completed 24-h urine collection both in summer and winter. In summer, 216 participants voided urine in the morning, 257 in the afternoon, 263 in the evening, and 268 overnight. In winter, 239 participants voided urine in the morning, and all of them urinated during other time periods. The baseline characteristics of the participants are shown in Table 1.

### 3.2. The Variability of 24-h Urine Collection in Different Seasons

The measured values of 24-h urine collection are shown in Table 2. The urinary sodium excretion of the participants was 3048.4 ± 1225.9 mg/day in summer and was 3564.7 ± 1369.6 mg/day in winter. The difference between the two seasons was statistically significant (*p* < 0.001).

### 3.3. The Bias between Mean Measured and Predicted 24-h Urinary Sodium Excretions (from Group Levels)

Mean predicted 24-h urinary sodium excretion varied by predictive methods, hours, and seasons (Table 3 and Table 4).

**Figure 1:**
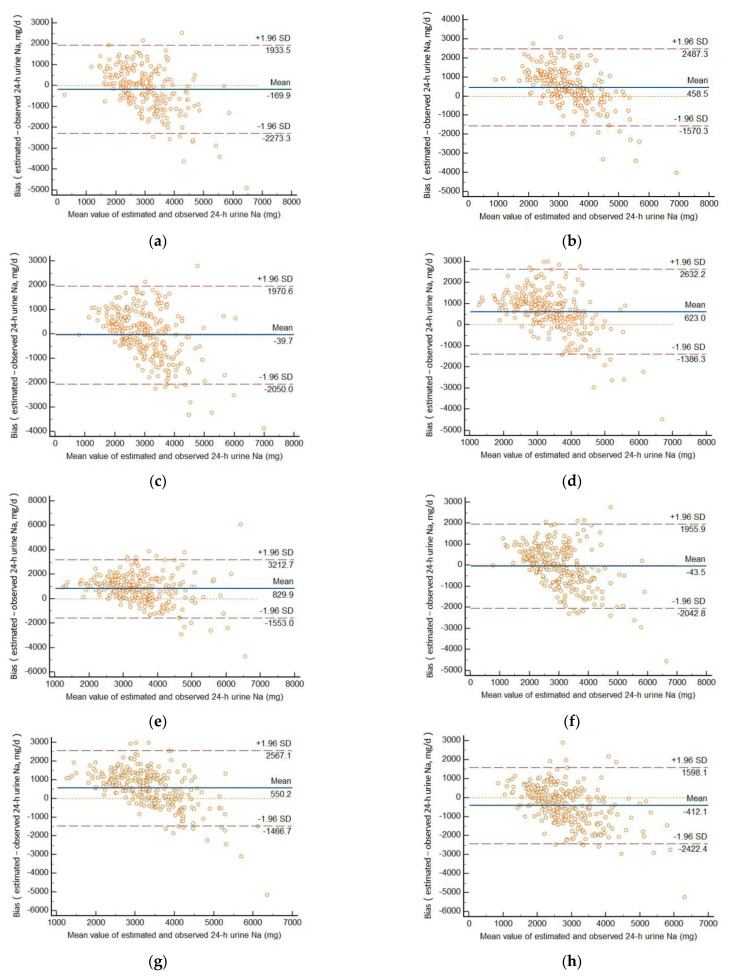

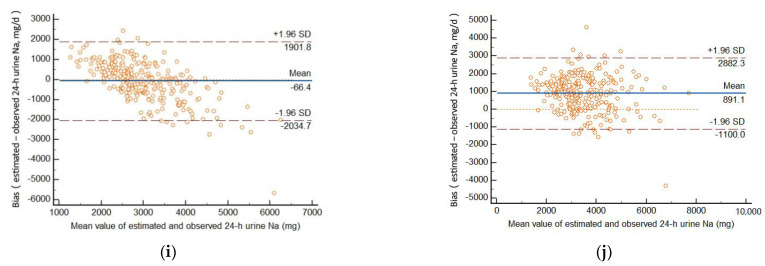
Bland–Altman plot of the bias (difference) between predicted and measured 24-h urinary sodium excretion in summer, based on INTERSALT equation (**a**) and Tanaka equation (**b**) in the morning, INTERSALT equation (**c**), Tanaka equation (**d**) and Sun’s equation (**e**) in the afternoon, INTERSALT equation (**f**) and Tanaka equation (**g**) in the evening, and INTERSALT equation (**h**), Tanaka equation (**i**) and Kawasaki equation (**j**) overnight.

In summer (Table 3, Figure 1), mean bias (predicted value—measured value) with the Kawasaki method was 891.1 mg (95% CI: 769.9, 1012.4 mg). Using the INTERSALT method, mean bias was small with morning samples (169.9 mg; 95% CI: −312.5, −27.3 mg), afternoon samples (−39.7 mg; 95% CI: −164.7, 85.3 mg), and evening samples (−43.5 mg; 95% CI: −166.4, 79.5 mg). With the Tanaka method, the bias was small with overnight samples (66.4 mg; 95% CI: −534.6, −289.7 mg). When used with the Sun’s method, mean bias was as high as 829.9 mg (95% CI: 681.7, 978.0 mg).

**Figure 2:**
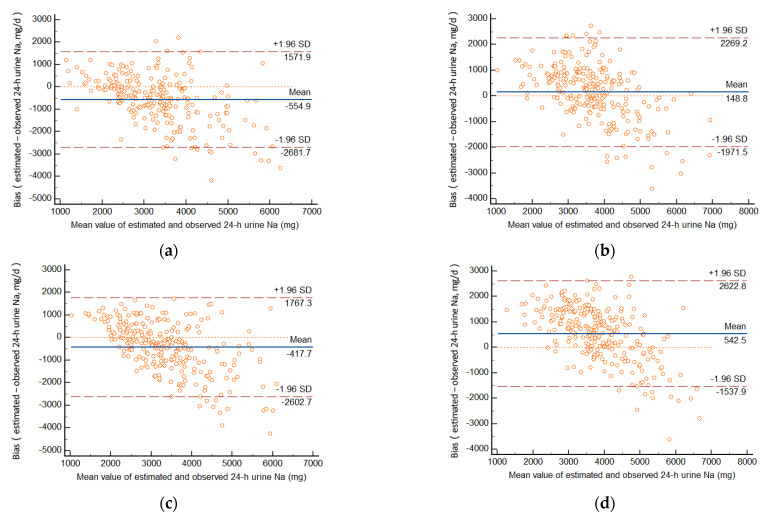

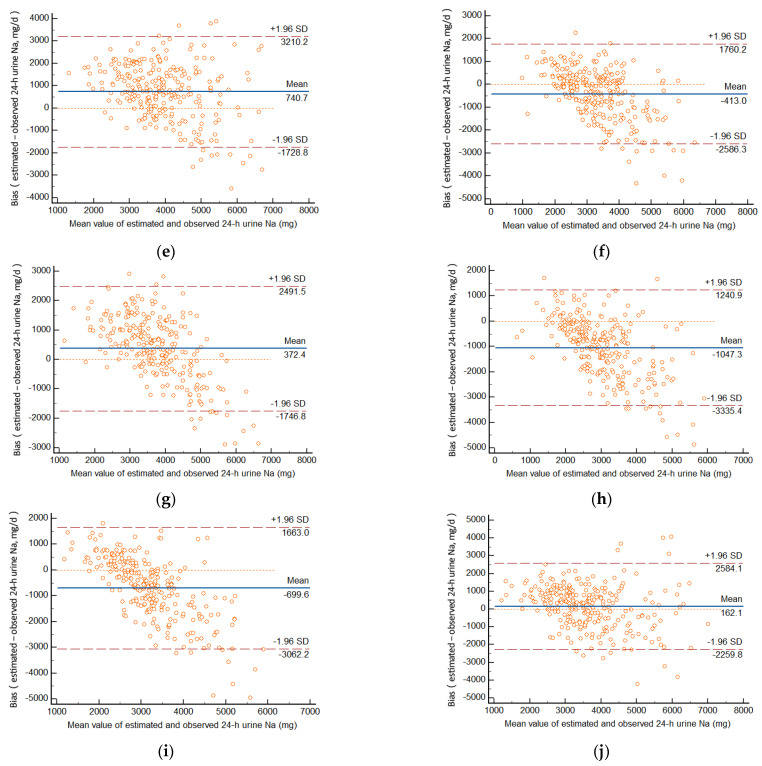
Bland–Altman plot of the bias (difference) between predicted and measured 24-h urinary sodium excretion in winter, based on INTERSALT equation (**a**) and Tanaka equation (**b**) in the morning, INTERSALT equation (**c**), Tanaka equation (**d**) and Sun’s equation (**e**) in the afternoon, INTERSALT equation (**f**) and Tanaka equation (**g**) in the evening, and INTERSALT equation (**h**), Tanaka equation (**i**) and Kawasaki equation (**j**) overnight.

In winter (Table 4, Figure 2), mean bias with the Kawasaki method was 162.1 mg (95% CI: 13.5, 310.7 mg), superior to other methods. With the Tanaka method, the bias was the smallest with the morning samples (148.8 mg; 95% CI: 11.0, 286.7 mg) in the morning, but 542.5 mg (95% CI: 414.8, 670.1 mg) in the afternoon, 372.4 mg (95% CI: 242.3, 502.4 mg) in the evening, and −699.6 mg (95% CI: −844.6, −554.6 mg) overnight. Using the Sun’s method, mean bias (740.7 mg; 95% CI: 589.2, 892.2 mg) was still higher than other methods, especially for women.

In both seasons, overestimation occurred with the Kawasaki, Tanaka (except overnight), and Sun’s method, while underestimation occurred with the INTERSALT method and Tanaka method used with the overnight samples.

### 3.4. Relative Individual Differences in Predicted and Measured 24-h Urinary Sodium Excretion

Bland–Altman plots show the relative individual differences between estimated values and measured values by 24-h urine collection. Figure 1 and Figure 2 indicate good agreement between predicted and measured values.

For individuals, in summer, among all estimated values, 4.5–5.1% of the cases exceeded the consistency boundary (±1.96 S) with the INTERSALT method, 4.1–5.8% exceeded the consistency boundary (±1.96 S) with the Tanaka method, 5.2% and 4.7% exceeded the consistency boundary (±1.96 S) with the Kawasaki and Sun’s method. In winter, the proportion of the cases exceeding the consistency boundary (±1.96 S) with the estimating methods was from 3.4% to 6.3%.

From Figure 1 and Figure 2, we can also find that the difference in predicted and measured 24-h urinary sodium excretion had a consistent trend. Overestimation appears to occur at low levels of 24-h sodium excretion and underestimation at high levels, no matter what season.

### 3.5. Individual Correlations with Measured 24-h Urinary Sodium Excretion

We compared the spot urine collections at all four times in two seasons with measured 24-h sodium excretions. The correlations were from 0.406 to 0.635 (Table 3 and Table 4). The lowest correlation with measured 24-h urinary sodium excretion was the Sun’s method both in summer (0.406) and winter (0.476). The Tanaka method used overnight samples in winter was 0.476, the same with the Sun’s method. The highest correlations were 0.612–0.635 when the Kawasaki method was used in summer (0.612) and the Tanaka method was used with afternoon (0.635) and evening (0.618) samples in winter.

## 4. Discussion

Our study used the spot urine collections at all four times (morning, afternoon, evening, and overnight) to estimate the sodium intake in summer and winter. According to the Bland–Altman plots, in summer, the INTERSALT method may provide the least biased information about group mean 24-h urinary sodium excretion. The mean biases were less than 50 mg with afternoon and evening samples. In winter, the Tanaka method with morning samples and the Kawasaki method were the most accurate for group mean 24-h urinary sodium excretion. For individuals, over- and underestimation occurred to different degrees. In general, overestimation appears to occur at low levels of 24-h urinary sodium excretion and underestimation at high levels.

The PURE study (multi-ethnic populations in 11 countries) [23], a China study among natural populations [24] and two studies among a population with hypertension [21,25] showed that the Kawasaki method was superior, which is similar to our study in winter. A US study found the INTERSALT method provided the least biased estimation [26]. That is consistent with our results in summer. Three previous studies in China concluded that the Tanaka method had the least bias in predicting population 24-h sodium excretion [27,28,29]. The reason for the discrepancy may be the difference in populations and time periods. The PURE study also illustrated that overestimation appears to occur at low levels of 24-h sodium excretion and underestimation at high levels [23].

We found that times and seasons of spot urine sample collection are key factors for the estimation of 24-h urinary sodium excretion. The temperature was an important factor for different seasons. Even when using the same method, biases were different between the two seasons. That may be caused by perspiration. Our results also showed that the daily urinary sodium excretion in summer was 520.7 mg (1.3 g synthetic salt) lower than the urinary sodium excretion in winter. Compared with summer collection, the winter collection had a higher volume. Thus, the urine collected in winter can better reflect the actual value than that in summer. We also found that results varied among different time points. Particularly when the INTERSALT and Tanaka methods were used, we can see that mean predicted 24-h urinary sodium excretion with overnight samples was lower than that with other times in a day. From the perspective of metabolism, among healthy people, most sodium is excreted in the afternoon and evening. From midnight to early morning, sodium excretion gradually decreases. In our study, the mean predicted 24-h urinary sodium excretion with afternoon and evening samples was also higher than that with morning samples. The US study indicated that mean predicted 24-h sodium excretion based on specimens collected in the afternoon and evening, compared with morning or overnight, was a better approximation of mean 24-h sodium excretion [26]. However, this study came to the conclusion only with the INTERSALT method.

The individual correlations (r = 0.406–0.635) of the four methods we used in estimating the 24-h urinary sodium excretion with spot urine samples fluctuated with the four methods. For individuals, the correlation coefficient with the gold standard of the Kawasaki method (0.612) was the highest in summer and that of the Tanaka method with the afternoon specimens (0.635) was higher in winter. The individual correlations were similar to previous studies. Kawasaki used the second urine sample in the morning of the Japanese population and obtained a correlation coefficient of 0.73 [19], Tanaka used the random urine samples of the Japanese population with a similar estimated method only obtained a correlation of 0.54 [20]. In the US study, all values estimated by time-point urine samples with prediction methods were moderately correlated with measured 24-h urinary sodium excretion, 0.40–0.60 [26]. Furthermore, the bias also fluctuated. Over- and underestimation occurred across low to high sodium levels. Therefore, these methods are not suitable for estimating individual 24-h sodium excretion.

The difference in the bias and correlations indicate that when using estimated methods to predict the 24-h sodium excretion with different random urine samples, it will fluctuate due to ethnic differences or the different times of urine collection. The amount of sodium intake may also be an important reason for the estimated deviation. It is possible that people in different countries with different sodium intakes need different methods to estimate their 24-h sodium intake [26]. There have long been controversies about different ways to assess sodium intake. The Kawasaki, INTERSALT, and Tanaka methods were created by foreign researchers. The Sun’s method, first proposed in China, created by Ningling Sun’s team, Peking University People’s Hospital, uses the spot urine during afternoon to predict 24-h urinary sodium excretion [21]. However, the participants in this study were the hypertension population. Hypertension may affect urinary sodium excretion, which makes its promotion limited. We used the method in our study, but it was not ideal. Further studies are needed to simulate new equations for healthy Chinese and to explore the best time for estimating the population’s mean sodium intake.

The novelty and strength of our study are as follows: We selected healthy volunteers from a medical university for the reason that medical college students have better compliance than the general population, and they come from multiple provinces. Furthermore, we assess the validity of the four methods at different times in summer and winter and try to find the reasons for the bias. Our study also had potential limitations. First, we just collected the 24-h urine for one day in each study. It is likely that the correlations observed would be different if they were collected on a different day. Second, we used the overnight urine sample instead of the second morning urine sample, which may result in a bias for the predicted sodium excretion with Kawasaki formulae. Finally, we purposefully selected participants aged from 18- to 25-year-old, and the sample size is small, which cannot represent the whole population in China. Thus, it is not known whether our results apply to younger or older persons and populations. We will explore the question in further study.

## 5. Conclusions

Estimation of sodium intakes using the four methods is affected by the time and temperature. In summer, the INTERSALT method may provide the best estimation of the population’s mean sodium intake. The Kawasaki method is superior to the other three methods for the population’s mean sodium intake in winter. When using the estimating methods to evaluate 24-h urinary sodium excretion, the impact of the season should be considered in the formula. Further research covering more Chinese population is needed to establish suitable predictive equations for evaluating mean 24-h urinary sodium excretion, which is an important part of the national nutrition survey for salt reduction.

## Figures and Tables

**Table 1:** Characteristics of participants by gender.

	Total (*n* = 268)	Male (*n* = 138)	Female (*n* = 130)
*n* (%)	268 (100)	138 (51.5)	130 (48.5)
Age (year)	19.9 ± 1.8	20.2 ± 1.9	19.5 ± 1.6
Weight (kg)	61.7 ± 13.4	68.9 ± 13.4	54.1 ± 8.4
Height (cm)	166.8 ± 8.7	172.8 ± 6.3	160.4 ± 5.9
WC ^1^ (cm)	73.3 ± 10.4	78.6 ± 10.3	67.7 ± 6.9
BMI ^2^ (kg/m^2^)	22.1 ± 3.7	23.1 ± 4.3	21.0 ± 2.7
SBP ^3^ (mmHg)	114.9 ± 14.0	123.3 ± 12.5	106.0 ± 9.1
DBP ^4^ (mmHg)	74.3 ± 8.1	77.0 ± 8.6	71.5 ± 6.6
Heart rate (bpm)	79.9 ± 12.9	78.7 ± 12.6	81.1 ± 13.2

^1^ WC, waist circumference; ^2^ BMI, Body Mass Index; ^3^ SBP, systolic blood pressure; ^4^ DBP, diastolic blood pressure.

**Table 2:** Results of 24-h urine measurement of college students by season.

	Summer	Winter	*t*	*p*
Urinary volume (mL)	1334.6 ± 654.5	1586.9 ± 655.1	−6.124	<0.001
Sodium excretion (mg/d)	3048.4 ± 1225.9	3564.7 ± 1369.6	−5.696	<0.001
Potassium excretion (mg/d)	1004.8 ± 364.2	1112.6 ± 357.6	−3.934	<0.001
Creatinine excretion (mg/d)	1285.1 ± 422.4	1294.6 ± 411.2	−0.466	0.642
Urinary Na/K (mg/mg)	3.2 ± 1.3	3.4 ± 1.4	−1.580	0.115

**Table 3:** Comparison of the average level of daily sodium intake estimated by the four methods with the actual measured values in summer.

	Total (mg/d)	Male (mg/d)	Female (mg/d)	Predicted Value—Measured Value (95%CI) (mg/d)	*r*	*p*	Probability That Estimated Value Exists Within Mean ± 1.96 SD
Measured 24-h sodium excretion	3043.0 ± 1223.3	3372.5 ± 1304.4	2688.3 ± 1201.1	Reference	—	—	Reference
Kawasaki	3934.2 ± 1058.5	4278.3 ± 1112.6	3563.7 ± 858.0	891.1 (769.9, 1012.4)	0.612	<0.001	5.2% 14/268
INTERSALT							
Morning	2934.9 ± 775.7	3387.0 ± 689.8	2535.3 ± 528.5	−169.9 (−312.5, −27.3)	0.537	<0.001	5.1% 11/216
Afternoon	3025.7 ± 801.0	3940.5 ± 752.5	2535.3 ± 504.0	−39.7 (−164.7, 85.3)	0.557	<0.001	4.7% 12/257
Evening	3016.7 ± 791.7	3462.0 ± 748.4	2547.4 ± 518.9	−43.5 (−166.4, 79.5)	0.559	<0.001	4.9% 13/263
Overnight	2630.9 ± 771.4	2983.0 ± 824.6	2251.8 ± 478.3	−412.1 (−534.6, −289.7)	0.551	<0.001	4.5% 12/268
Tanaka							
Morning	3563.4 ± 712.2	3678.6 ± 689.6	3441.7 ± 718.6	458.5 (321.0, 596.0)	0.577	<0.001	4.6% 10/216
Afternoon	3688.4 ± 739.1	3720.4 ± 722.5	3654.6 ± 757.5	623.0 (498.0, 747.9)	0.552	<0.001	5.8% 15/257
Evening	3610.3 ± 676.6	3682.4 ± 685.7	3534.3 ± 660.9	550.2 (426.2, 674.2)	0.541	<0.001	4.9% 13/263
Overnight	2976.6 ± 626.8	3038.6 ± 653.7	2909.8 ± 591.9	−66.4 (−186.3, 53.4)	0.574	<0.001	4.1% 11/268
Sun’s	3895.3 ± 966.5	3768.7 ± 938.6	4028.8 ± 981.2	829.9 (681.7, 978.0)	0.406	<0.001	4.7% 12/257

**Table 4:** Comparison of the average level of daily sodium intake estimated by the four methods with the actual measured values in winter.

	Total (mg/d)	Male (mg/d)	Female (mg/d)	Predicted Value—Measured Value (95%CI) (mg/d)	*r*	*p*	Probability that Estimated Value Exists Within Mean ± 1.96 SD
Measured 24-h sodium excretion	3563.7 ± 1370.0	4034.0 ± 1354.6	3062.3 ± 1190.6	Reference	—	—	Reference
Kawasaki	3726.3 ± 1086.9	4160.9 ± 1141.6	3265.1 ± 803.0	162.1 (13.5, 310.7)	0.515	<0.001	4.5% 12/268
INTERSALT							
Morning	3084.7 ± 863.2	3591.6 ± 788.6	2519.1 ± 546.7	−554.9 (−693.2, −416.6)	0.597	<0.001	5.0% 12/239
Afternoon	3144.3 ± 808.3	3583.6 ± 775.1	2678.1 ± 537.4	−417.7 (−551.7, −283.6)	0.581	<0.001	4.1% 11/268
Evening	3149.2 ± 831.0	3665.0 ± 689.0	2601.8 ± 0570.3	−413.0 (−546.4, −279.7)	0.587	<0.001	4.1% 11/268
Overnight	2515.9 ± 755.1	2673.7 ± 854.3	2340.2 ± 595.8	−1047.3 (−1187.7, −906.9)	0.524	<0.001	4.5% 12/268
Tanaka							
Morning	3753.1 ± 830.3	4011.4 ± 816.6	3501.2 ± 766.5	148.8 (11.0, 286.7)	0.599	<0.001	6.3% 15/239
Afternoon	4105.8 ± 782.6	4196.7 ± 751.2	4009.3 ± 806.2	542.5 (414.8, 670.1)	0.635	<0.001	4.5% 12/268
Evening	3939.7 ± 745.1	4016.9 ± 663.3	3857.8 ± 817.8	372.4 (242.3, 502.4)	0.618	<0.001	5.2% 14/268
Overnight	2864.8 ± 610.0	2901.8 ± 661.1	2825.8 ± 557.1	−699.6 (−844.6, −554.6)	0.476	<0.001	3.4% 9/268
Sun’s	4309.1 ± 1026.0	4229.3 ± 934.1	4364.0 ± 1105.1	740.7 (589.2, 892.2)	0.476	<0.001	4.9% 13/268

## Data Availability

Not applicable.

## Supplementary Materials

The following supporting information can be downloaded at: https://www.mdpi.com/article/10.3390/nu14132736/s1, Table S1: Predictive methods for 24-h sodium excretion based on single spot urinary sodium concentrations.
