# Supplementary material for: Validation of 4 Estimating Methods to Evaluate 24-h Urinary Sodium Excretion: Summer and Winter Seasons for College Students in China
Source: Nutrients. 2022 Jun 30;14(13):2736. doi: 10.3390/nu14132736 (PMC9269089; doi:10.3390/nu14132736)
Supplement: Supplementary file 1 [file nutrients-14-02736-s001.zip › nutrients-1766075-supplementary.pdf]

TableS1. Predictive methods for 24-h sodium excretion based on single spot urinary sodium concentrations

Method	Urine sample	Predictive method for 24-h sodium excretion
Kawasaki	The second morning urine sample1	Estimated sodium (mEq/day) = 16.3 × [spot Na (mEq/L)/spot Cr (mg/dL × 10) × Pr24 hCr (mg/day)]0.500<br>Male: Pr24 hCr (mg/day) = 15.12 × weight (kg) + 7.39 × height (cm)−12.63 × age (years) −79.9<br>Female: Pr24 hCr (mg/day) = 8.58 × weight (kg) + 5.09 × height (cm) −4.72 × age (years) −74.95
INTERSALT	Spot urine sample	Male: 24h Estimated sodium (mg/day) = 23 × [25.46 + 0.46 × spot Na (mmol/L) −2.75 × spot Cr (mmol/L)−0.13 × spot K (mmol/L) + 4.10 × BMI (kg/m2) + 0.26 × age (years)]<br>Female: 24h Estimated sodium (mg/day) =23×[5.07+0.34 × spot Na (mmol/L)−2.16 × spot Cr (mmol/L)−0.09 × spot K (mmol/L) + 2.39 × BMI (kg/m2) + 2.35 × age (years) −0.03 ×age (years)2]
Tanaka	Spot urine sample	24h Estimated sodium (mg/day) = 23 × 21.98 × { spot Na (mEq/L) / [spot Cr (mg/ dL) × 10] × Pr24 hCr (mg/day)}0.392<br>Pr24hCr (mg/day) = 14.89 × weight (kg) + 16.14 × height (cm) −2.04 × age (years)−2244.45
Sun's	Afternoon spot urine sample	Male: 24h Estimated sodium (mg/day) = 0.208 × Pr24hCr (mg/day) × [spot Na (mmol/L) / spot Cr (mg/ L)]0.300 × 23<br>Pr24hCr (mg/day) = e6.916−0.007 × age (years) −0.002 × height (cm) + 0.013 × weight (kg)<br>Female: 24h Estimated sodium (mg/day) = 0.329 × Pr24hCr (mg/day) × [spot Na (mmol/L) / spot Cr (mg/ L)]0.463 × 23<br>Pr24hCr (mg/day) = e7.210−0.013 × age (years) −0.002 × height (cm) + 0.010 × weight (kg)

*We used the overnight urine sample instead of the second morning urine sample in this studay.

Pr24hCr, predicted 24-h urine creatinine excretion.

BMI, body mass index.
